# Arrhythmic Song Exposure Increases ZENK Expression in Auditory Cortical Areas and Nucleus Taeniae of the Adult Zebra Finch

**DOI:** 10.1371/journal.pone.0108841

**Published:** 2014-09-26

**Authors:** Jennifer Lampen, Katherine Jones, J. Devin McAuley, Soo-Eun Chang, Juli Wade

**Affiliations:** 1 Neuroscience Program, Michigan State University, East Lansing, Michigan, United States of America; 2 Department of Psychology, Michigan State University, East Lansing, Michigan, United States of America; 3 Department of Psychiatry, University of Michigan, Ann Arbor, Michigan, United States of America; Texas Christian University, United States of America

## Abstract

Rhythm is important in the production of motor sequences such as speech and song. Deficits in rhythm processing have been implicated in human disorders that affect speech and language processing, including stuttering, autism, and dyslexia. Songbirds provide a tractable model for studying the neural underpinnings of rhythm processing due to parallels with humans in neural structures and vocal learning patterns. In this study, adult zebra finches were exposed to naturally rhythmic conspecific song or arrhythmic song. Immunohistochemistry for the immediate early gene ZENK was used to detect neural activation in response to these two types of stimuli. ZENK was increased in response to arrhythmic song in the auditory association cortex homologs, caudomedial nidopallium (NCM) and caudomedial mesopallium (CMM), and the avian amygdala, nucleus taeniae (Tn). CMM also had greater ZENK labeling in females than males. The increased neural activity in NCM and CMM during perception of arrhythmic stimuli parallels increased activity in the human auditory cortex following exposure to unexpected, or perturbed, auditory stimuli. These auditory areas may be detecting errors in arrhythmic song when comparing it to a stored template of how conspecific song is expected to sound. CMM may also be important for females in evaluating songs of potential mates. In the context of other research in songbirds, we suggest that the increased activity in Tn may be related to the value of song for assessing mate choice and bonding or it may be related to perception of arrhythmic song as aversive.

## Introduction

Human speech and avian song have many parallels: both are acquired through sensorimotor learning, and when well-formed they are rhythmically structured in time. There is increasing evidence that rhythm plays an important role in speech and language processing. During development, rhythm perception ability is positively correlated with language and literacy skill [1]. Moreover, children with specific language impairment (language delay) have deficits in rhythm processing that include the ability to move in synchrony with a beat [2], [3]. People who stutter also appear to have deficits in internal rhythm generation and timing for speech, but can produce fluent speech when paced by an external rhythm such as a metronome [4], another speaker [5], or singing [6]. A number of other human disorders also involve disruptions in timing and/or rhythm processing. For example, individuals with autism have been proposed to show deficits in temporal processing [7], with presentation of auditory rhythms possibly alleviating some symptoms [8]. Disruptions in aspects of timing or rhythm processing have also been observed in patients with attention deficit hyperactivity disorder (reviewed in [9]), schizophrenia (reviewed in [9]), and dyslexia [10]. Parkinson’s disease patients also show significant impairment in rhythm perception [11]. Thus, a better understanding of the neural bases of rhythm processing could elucidate mechanisms associated with a wide range of human developmental and psychiatric disorders.

**Figure 1:**
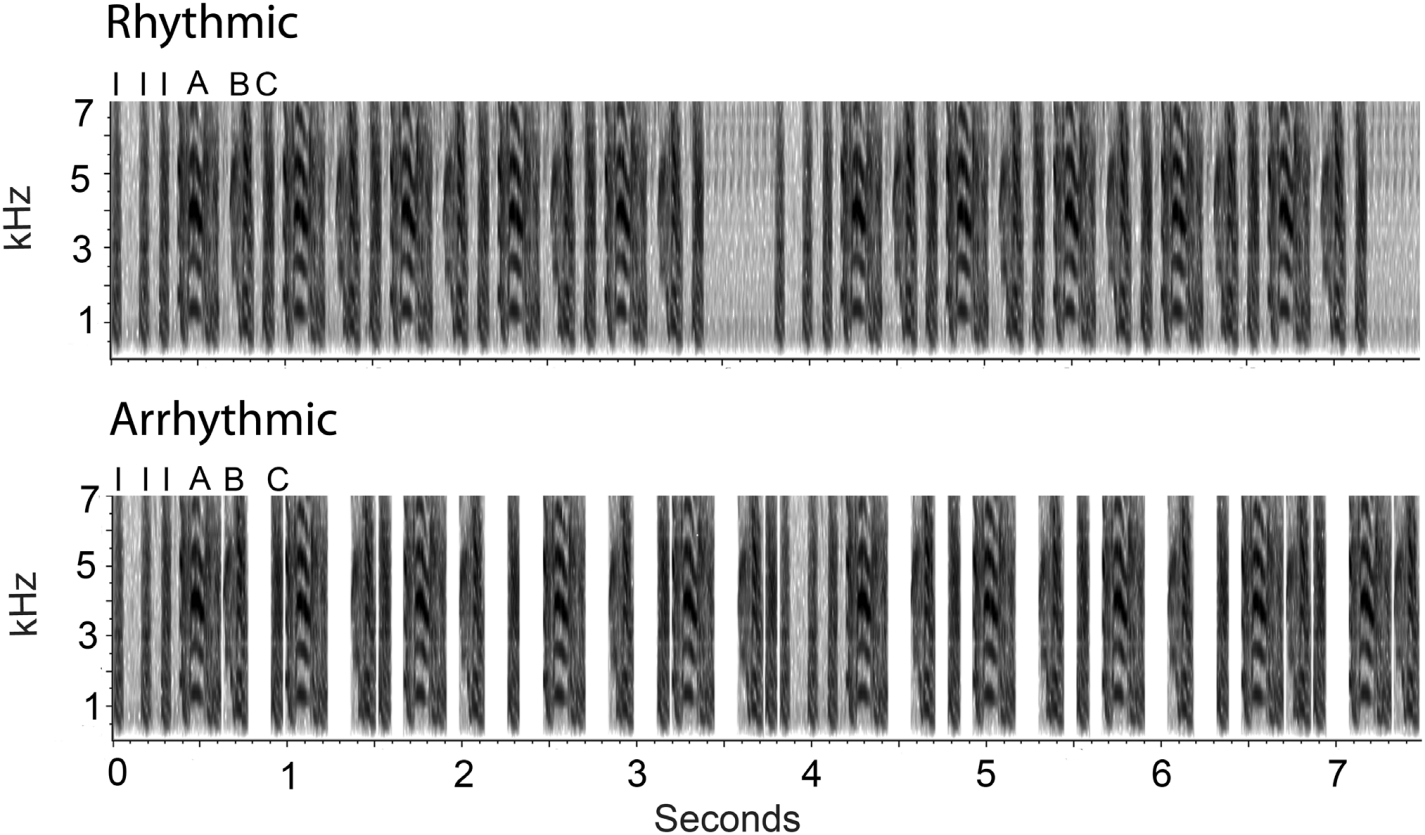
Representative spectrograms of rhythmic and arrhythmic song. Images depict 7.5 seconds of representative rhythmic and arrhythmic song stimuli. They were generated from the same natural song. Introductory notes are indicated with I. A, B, and C indicate 3 distinct notes that compose a motif. Each image contains two bouts of song.

Zebra finches represent an excellent potential model for studying neural mechanisms of timing and rhythm perception. As songbirds, they produce highly rhythmic vocalizations used for courtship and the defense of nest sites [12]. Zebra finch song begins with a series of short introductory notes, followed by several repetitions of an ordered set of notes called a motif (Figure 1). A complete sequence of introductory notes and subsequent motifs performed without a prolonged silent interval is referred to as one song bout. The intervals between notes are very regular. This consistent natural rhythm of zebra finch song [12] provides a relatively unique opportunity to study rhythm as a discriminatory characteristic. Furthermore, as an animal model, zebra finches provide an opportunity to study the neural basis of rhythm perception in a more direct manner than possible with humans.

Zebra finches are vocal learners [13] and, similarly, normal human rhythm processing has been proposed to be a by-product of vocal learning mechanisms [14]. Moreover, as vocal learners, zebra finches learn to sing in a manner similar to the way humans develop speech (reviewed in [13]). Both species initially form an auditory template by listening to vocal production of adult tutors. They then practice and improve on their own vocalizations, which include subsong in birds and babbling in humans, and ultimately produce adult crystalized song in zebra finches and fluent speech in humans. In both species, critical periods exist after which vocal learning is strongly limited. In addition to the similar developmental trajectories, humans and zebra finches have substantial parallels in the neural structures underlying the perception, learning, and production of vocalizations [15]. Area X, part of an anterior circuit involved in song learning, is not visible in female zebra finches [16], [17] who do not sing, and is homologous to the human striatum [18], [19] which is involved in language learning [20]. HVC (proper name) is similar to the premotor cortex [21] and the robust nucleus of the arcopallium (RA) is similar to the motor cortex in humans [22]. Both HVC and RA are part of a motor circuit involved in song production. Both of these areas are larger in male than in female zebra finches [16], [17]. Although only male zebra finches sing, females likely also acquire a song template from their fathers [23]–[25], which is presumably used as a model for quality in the selection of potential mates [12], [26], [27]. The caudomedial nidopallium (NCM) and caudomedial mesopallium (CMM), while anatomically distinct in the zebra finch brain, are both considered homologous to the auditory association cortex in humans [28]. The lateral magnocellular nucleus of the anterior nidopallum (LMAN) is part of the circuit essential for song learning, and is necessary for song plasticity during development [29]–[31] and in adulthood [32]–[34]. A homologous region to LMAN within the human brain has not been identified.

Many studies have investigated zebra finch auditory perception and the factors influencing neural responses to auditory stimuli. A marker commonly used to assess neural activation in zebra finches is the immediate early gene ZENK [35]–[43]. ‘ZENK’ is an acronym used to identify the evolutionally conserved protein based on names from other species, specifically zif-268 [44], egr-1 [45], NGFI-A [46], and Krox-24 [47]. The ZENK protein has a DNA binding site and can regulate the expression of other genes [48]. It is thought to be involved in synaptic plasticity, and memory [49]. Extracellular signal-related kinase is an upstream component of the signaling pathway including ZENK; its inhibition blocks induction of ZENK in the zebra finch auditory cortex [38]. If the pathway for ZENK induction is interrupted in juvenile zebra finches, song learning is significantly reduced [50].

Both male and female zebra finches show robust induction of ZENK in multiple brain regions in response to conspecific song [36]. For example, NCM and CMM express high levels of ZENK following zebra finch song playbacks [40]–[42]. Investigations have also repeatedly demonstrated increased ZENK with presentation of conspecific compared to heterospecific song, pure tones, or silence in both adults [42], [51] and juveniles [35], [36]. The increase in neural activity in response to conspecific song has been demonstrated using other methodologies such as fMRI [52], [53], and electrophysiology [54]. ZENK expression in NCM and CMM is also greater in response to song from tutored compared to untutored zebra finch males [43], [55]. Immediate early gene expression following tutor song presentation is significantly correlated with the strength of song learning [56], [57]. Together these results suggest that ZENK expression in auditory cortical regions is highest in response to stimuli that are most similar to the song template learned during development.

Distinct patterns of ZENK expression suggest that specific brain regions are activated by *hearing* song and others are associated with *producing* it [39], [41]. In canaries, singing induces ZENK expression in HVC, RA, LMAN, and Area X, among other nuclei [39]. Hearing conspecific song creates a different pattern of ZENK expression in canaries, with abundant expression in portions of the primary auditory cortex homolog field L, as well as CMM and NCM [39]. Parallel patterns of ZENK expression are seen in zebra finches that sing or hear conspecific song [41]. In zebra finches, HVC also shows significant electrophysiological responses to a bird’s own song in anesthetized subadults [58] and sleeping juveniles, and tutor’s song in awake juveniles [59], with much less response to other conspecific songs. However, the same pattern has not been described in ZENK responses [37] when assessed in awake adults.

While many aspects of zebra finch auditory responses have been studied, little is known about their perception of rhythm, and whether rhythm is a salient factor in their discrimination of songs. To evaluate whether rhythmicity influences neural responses, and which brain regions are involved in processing information about rhythmic structure in zebra finches, the present study exposed adult males and females to conspecific songs with normal structure (‘rhythmic’) or the same vocalizations with varied timing of syllable onset while maintaining the same syllable order (‘arrhythmic’). The density of ZENK immunoreactive cells was assessed in several brain areas of interest, including regions important to song production and perception. Expression was also quantified in nucleus taeniae (Tn; a homolog to the mammalian amygdala, [60]), because an initial qualitative assessment indicated particularly strong labeling there. We hypothesized that ZENK expression would differ in birds exposed to rhythmic or arrhythmic song in nuclei involved in the perception or evaluation of auditory stimuli.

## Materials and Methods

### Animals

Zebra finches were raised in walk-in aviaries at Michigan State University, each containing 5–7 pairs of males and females with their offspring. Birds were maintained on a 12∶12 light:dark cycle with lights turning on at 7 am and provided *ad libitum* access to seed (Kaytee Finch Feed, Chilton, WI), water, gravel and cuttlebone. Their diet was supplemented weekly with hard boiled chicken eggs mixed with bread, as well as spinach and oranges. Once birds reached adulthood (at least 90 days of age), they were transferred to adjacent single sex walk-in aviaries and allowed a minimum of 10 days to acclimate to their new housing prior to experimental stimulus exposure. Birds could see and hear birds of the opposite sex, but could not physically interact. Animals in the study were less than 1 year old.

### Ethics Statement

All protocols were approved by the Institutional Animal Care and Use Committee of Michigan State University (#01-13-006-00).

### Stimulus Creation

Nine 30-second rhythmic song stimuli and nine 30-second arrhythmic song stimuli were formed using Praat software [61] (Figure 1). To create these stimuli, nine zebra finch song recordings were selected from Boston University’s Laboratory of Neural Circuit Information Zebra Finch song data set (http://people.bu.edu/timothyg/song_website/index.html). For each stimulus, introductory syllables and two subsequent motifs (motif 1 and motif 2) were extracted from a recording. They were alternated 5 times, forming an alternating (1-2-1-2-1) motif structure. Thus a single bout of a song consisted of an unmanipulated sequence of introductory notes, followed by five unmanipulated motif productions (Figure 1). To form a complete rhythmic song stimulus, bouts were repeated for 30 seconds, with at least 0.4 seconds of silence between each bout presentation. The remaining silence, after repeating bouts until a complete bout could not be repeated without surpassing 30 seconds, was distributed evenly across the intervals between bouts so that each complete stimulus was 30 seconds. Across the rhythmic stimuli, silence between bouts ranged from 0.4 to 1.4 seconds (mean silence between bouts = 0.8 seconds). The 9 rhythmic song stimuli were divided into 3 groups of 3 such that the total length of silence was similar across groups. Maintaining a similar amount of total auditory stimulus across groupings was important because duration of song exposure can influence levels of ZENK expression [40].

To create the nine arrhythmic song stimuli, the length of each interval between syllables (other than between introductory notes), motifs, and bouts of the rhythmic song stimuli was altered using Matlab (The Mathworks, Inc., Natick, MA). The same total amount of spacing within the 30-second stimulus was retained. However, each interval was randomly changed to one of three durations: 1) 10 ms, 2) the average duration (based on all intervals in a song except those between introductory notes), or 3) double the average duration, minus 10 ms. After all but the final interval had been changed to one of those three durations, the final one in each song was changed to the duration needed to add up to the original total (Figure 1). In this manner, the sequential order of syllables was preserved, but the rhythmicity, or regularity, of the timing of the syllables was disrupted, yielding arrhythmic songs. The 9 arrhythmic song stimuli were divided into 3 groups of 3 corresponding to the same grouping as the rhythmic stimuli. There were a total of 6 groups, 3 in the rhythmic condition, and 3 in the arrhythmic condition.

### Song Exposure

For each stimulus type, 9 males and 9 females were exposed. Presentation of stimuli was controlled using E-Prime 2.0 software (Psychology Software Tools, Inc., Pittsburgh, PA). Individual birds were exposed to a stimulus inside an 11.25″×8.5″×15.25″ cage within a 7′2″×14′9″ room with lights on. First, a 1-hour period of silence allowed birds to acclimate to the testing room. Following the hour of silence, each bird was exposed to one group of 3 songs (either rhythmic or arrhythmic, randomly selected). Songs were played from a single speaker adjacent to the testing cage. Song stimuli were presented in pseudo-random order for a total of 30 presentations (10 presentations of each 30-second song), yielding a total of approximately 15 minutes of song. For every 3 presentations, each song was heard once. There were 30 seconds of silence between each song. Therefore, the song presentation portion of the procedure lasted approximately 30 minutes. All songs were played within the volume range of normal zebra finch song, at approximately 70 dB. Both the testing room and all stimuli were novel for all birds. All testing occurred between 9 am and 3 pm, with a maximum of two birds tested in a day. Different stimulus groups were randomized across morning and afternoon testing times. Following song exposure animals remained in the testing room for 1 hour undisturbed in order to allow ZENK protein expression to reach peak levels [41]. They were then euthanized by rapid decapitation, whole brains were collected and frozen in methylbutane. Brains were stored at −80°C until further processing.

All of the song exposures were recorded using a Canon Vixia HF R300 camcorder. Recordings of all of the males were reviewed to ensure that the bird did not sing in response to the song presentation, as this could lead to a different pattern of ZENK expression in the brain than auditory song exposure alone [39], [41]. No males sang. Across both stimulus conditions, birds generally responded to the initiation of song by adopting an upright, alert posture, orienting toward the speaker, and some emitted a few chirps. All recordings were reviewed to determine whether excess background noise was present. Two birds were eliminated from analysis due to the presence of substantial, unanticipated noise near the testing room. A few other animals were eliminated from analysis of individual brain regions due to histological artifact. Final sample sizes are indicated in the figures.

### Tissue Processing

Brains were coronally sectioned at 20 µm and thaw mounted onto SuperFrost Plus slides (Fisher Scientific, Hampton, NH) in 6 series. Tissue was stored at −80°C until further processing. One series of slides was processed using immunohistochemistry for ZENK. Tissue was run in three groups due to the large number of slides; both sex and stimulus conditions were equally represented in each. Tissue was warmed to room temperature then rinsed in 0.1 M phosphate-buffered saline (PBS). It was then fixed in 4% paraformaldehyde, rinsed 3×5 minutes with PBS, and treated for 30 minutes with 0.9% H_2_O_2_ in methanol. It was rinsed with PBS, and incubated in 5% normal goat serum in PBS with 0.3% Triton X-100 for 30 minutes. Next, it was incubated overnight in a ZENK (Egr-1) rabbit polyclonal antibody (0.5 µg/ml, sc-189, Santa Cruz Biotechnology, Inc., Dallas, TX) in 5% normal goat serum in PBS with 0.3% Triton X-100 at 4°C. The tissue was rinsed in PBS and exposed to a biotin-conjugated goat anti-rabbit secondary antibody (0.5 µg/ml; Vector Labs, Burlingame, CA) in PBS with 0.3% Triton X-100 for 2 hours at room temperature. Following PBS rinses, it was incubated in Elite ABC reagents (Vector Labs, Burlingame, CA) for 1 hour, washed with PBS and Tris-buffered saline and then treated with diaminobenzadine in tris-buffered saline with 0.003% H_2_O_2_ to produce a brown reaction product. The reaction was terminated with PBS, and the tissue was dehydrated and coverslipped with DPX (Sigma–Aldrich, St. Louis, MO).

An adjacent series was stained with thionin to allow confirmation of the location of the brain regions of interest: NCM, CMM, Tn, lateral and medial striatum, HVC, and LMAN. The auditory cortical regions NCM and CMM were selected due to their role in song learning and perception. The telencephalic song control nuclei, striatum, HVC and LMAN, were selected due to their role in song learning and song production. Tn was selected because it is involved in motivated behaviors in birds [60], and on initial inspection of the tissue it showed high levels of ZENK expression.

**Figure 2:**
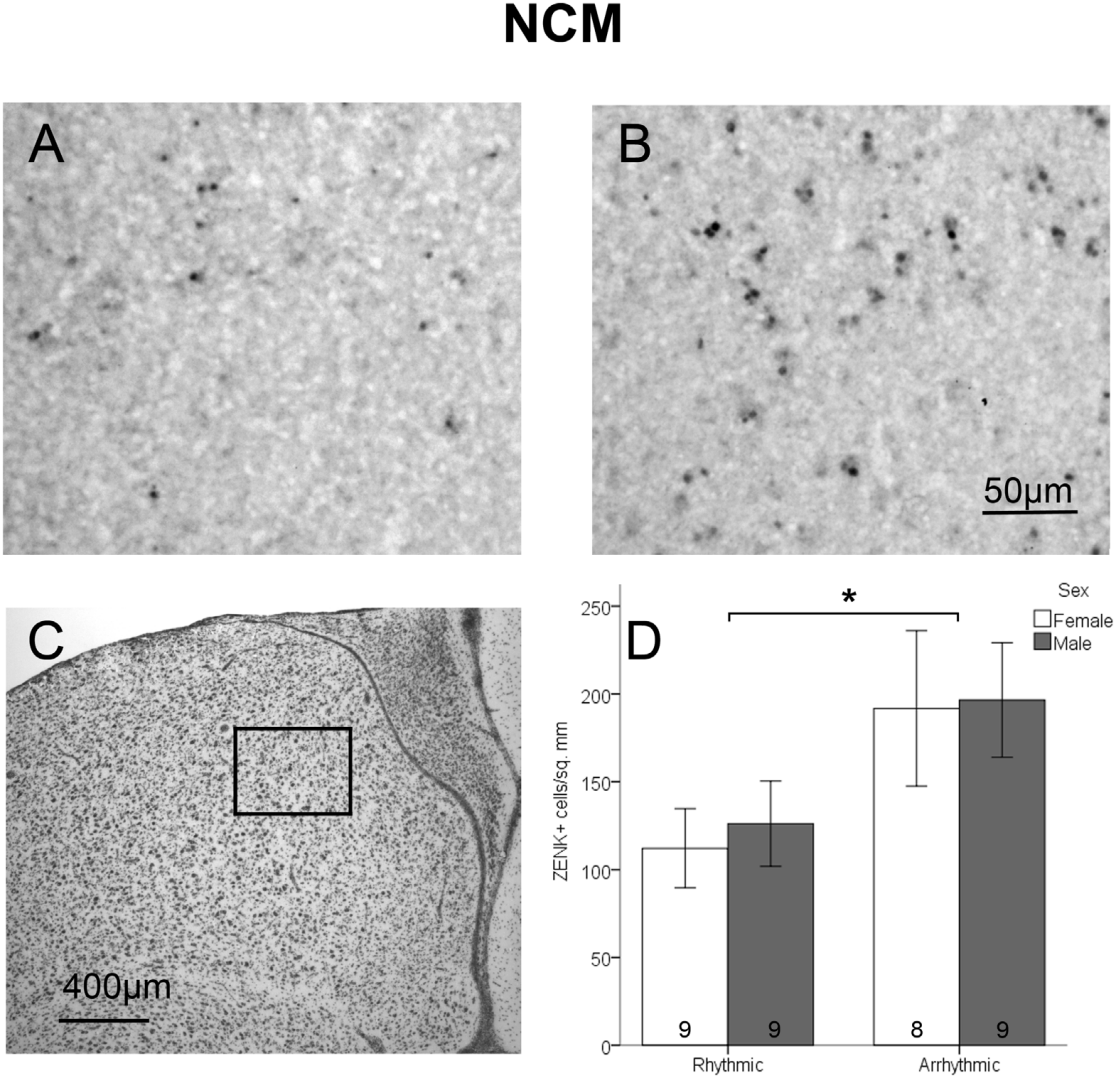
Density of ZENK expressing cells in NCM. Panels A and B depict representative samples of ZENK immunohistochemical labeling in birds exposed to rhythmic (A) or arrhythmic (B) song. Panel C depicts an adjacent section stained with thionin; the box indicates the area where cells were counted. Panel D shows the density of ZENK expressing cells between sexes and stimulus types (mean ± standard error). There was a significant main effect of sex, indicted by an asterisk. Sample sizes are noted within the bars.

**Figure 3:**
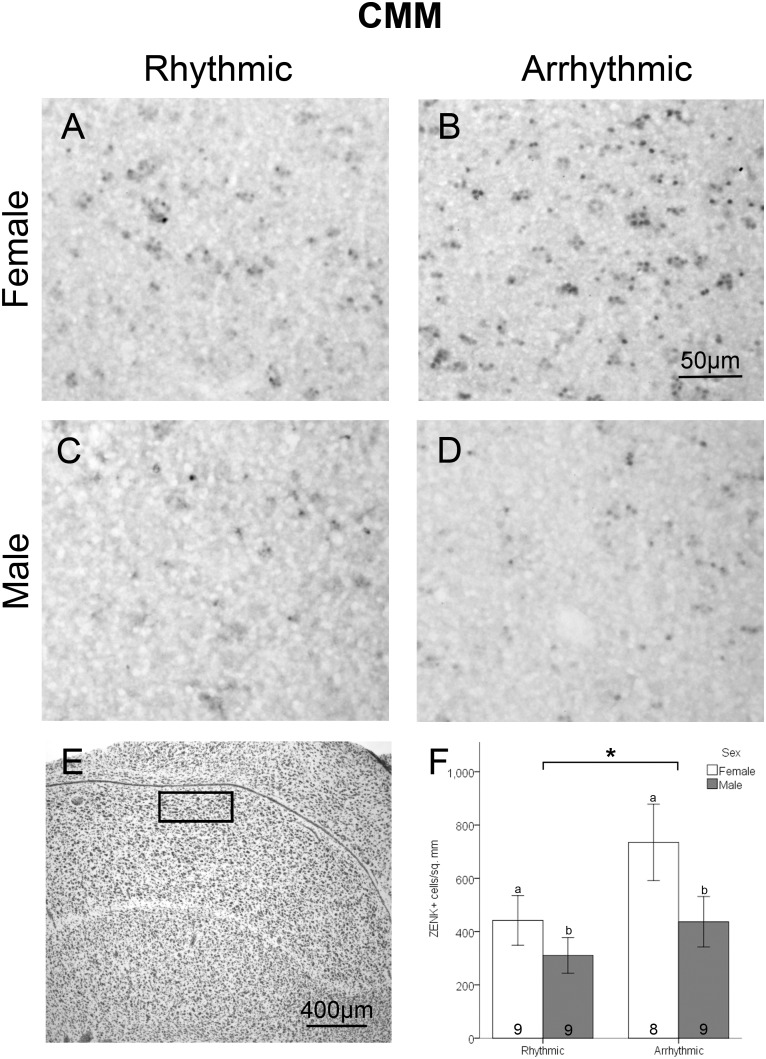
Density of ZENK expressing cells in CMM. Panels A-D depict representative samples of ZENK immunohistochemical labeling in a female exposed to rhythmic song (A), female exposed to arrhythmic song (B), male exposed to rhythmic song (C), and a male exposed to arrhythmic song (D). Panel E depicts a thionin stained adjacent section; the box indicates the area where cells were counted. Panel F depicts the density of ZENK expressing cells between sexes and stimulus types (mean ± standard error). A significant main effect of stimulus type is indicated by the asterisk. A significant main effect of sex is represented by the different lower case letters. Sample sizes are noted within the bars.

**Figure 4:**
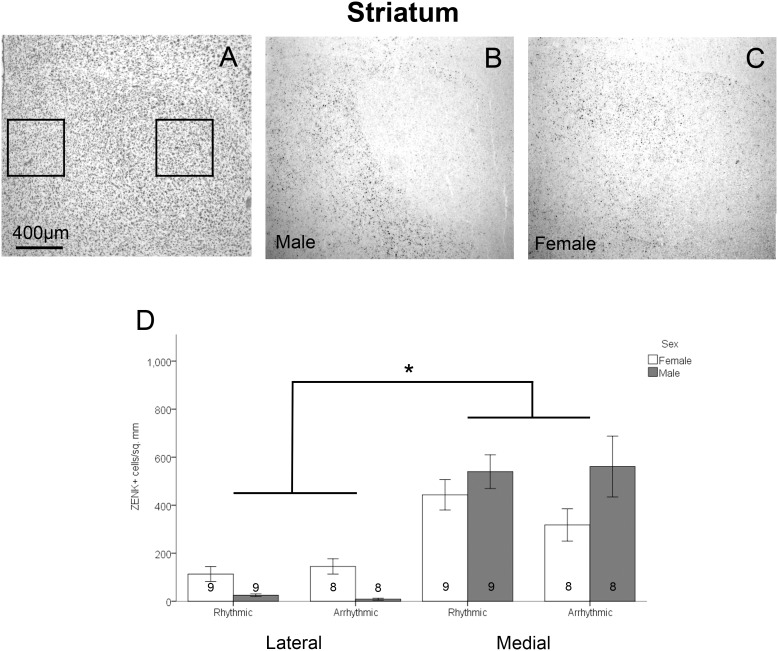
Density of ZENK expressing cells in the striatum. Panel A depicts a thionin stained section, with boxes showing the lateral and medial areas in which cell densities were assessed. Panel B is from a representative male exposed to rhythmic song, and C is from a female exposed to arrhythmic song. Panel D depicts the density of ZENK expressing cells between sexes, stimulus types, and location within the striatum (mean ± standard error). A main effect of location is indicated by the asterisk. A significant sex×region interaction was also detected, such that the difference in density of ZENK expressing cells in the medial compared to lateral striatum was greater in males than females. Sample sizes are noted within the bars.

**Figure 5:**
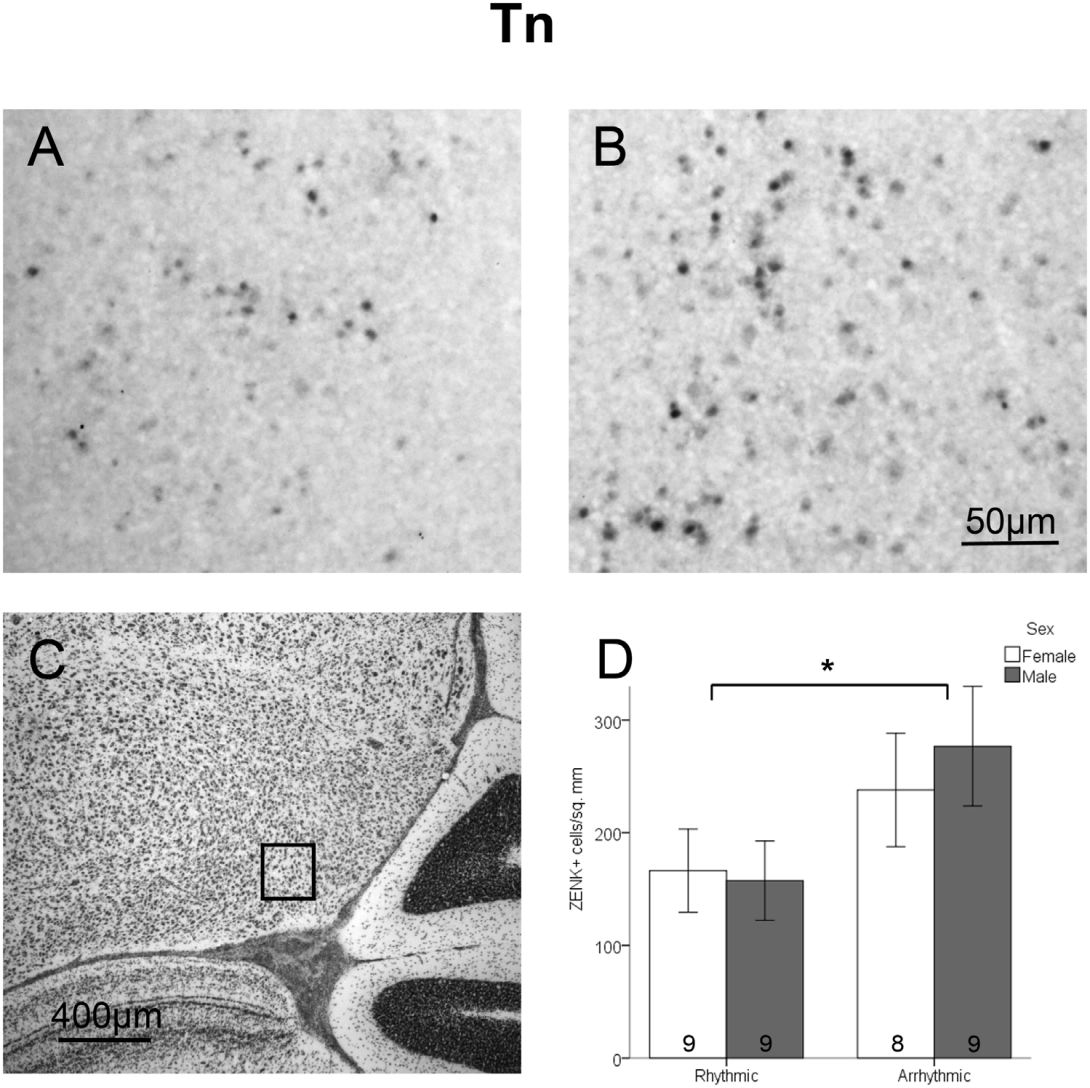
Density of ZENK expressing cells in Tn. Panels A and B depict representative samples of ZENK immunohsitochemical labeling in birds exposed to rhythmic (A) or arrhythmic (B) song. Panel C depicts a thionin stained adjacent section; the box indicates where cells were counted. Panel D shows the density of ZENK expressing cells between sexes and stimulus types (mean ± standard error). A significant main effect of sex is indicted by the asterisk. Sample sizes are noted within the bars.

Analysis of tissue sections was conducted by an observer blind to treatment condition and sex, using ImageJ software (National Institutes of Health). Each brain region was assessed bilaterally in two adjacent sections in each animal. For all brain regions assessed, a cell was considered labeled if it contained a round nuclear area densely filled with brown stain which was darker than the general background coloring seen in surrounding areas. For NCM, a 0.525 mm*0.393 mm box was placed with the medial corner under the hippocampus at the point where the ventricle begins to curve ventrally to run parallel with the midline (Figure 2C). A grid of 0.066 mm*0.065 mm rectangles existed within the box, and cells were counted in alternating cells of the grid excluding cells that overlapped with the bottom or left edge of each grid box. Density was determined by dividing the total number of labeled cells by half of the total area of the region analyzed. Cells within NCM were counted in the section prior to the start of RA and the first section containing RA. For CMM, a 0.496 mm*0.205 mm box was placed under the ventricle lateral to where it curves ventrally toward the midline between A 1.6 and A 1.2 from a songbird brain atlas [62] (Figure 3E). Area X is located in the lateral striatum of males, but is typically not visible in females [63]. Initial observations indicated substantial differences in the patterns of ZENK expression between the medial and lateral striatum. A box of 0.492 mm*0.492 mm was placed in the lateral portion of the medial striatum. For the medial striatum, a box of the same size (0.492 mm*0.492 mm) was placed half way between the midline and the location quantified in the lateral striatum (Figure 4A). Labeled cells were counted in striatum sections starting in the 4^th^ section after the appearance of LMAN in order to maintain a landmark that was visible in both sexes. For Tn, a 0.238 mm*0.244 mm box was placed near the ventral edge of the telencephalic lobe where a corner is formed by the ventral and medal edges of the lobe (Figure 5C). As with NCM, cells in Tn were counted in the section prior to the start of RA and the first section containing RA.

Limited labeling was detected in HVC and LMAN, so it was not quantified. However, a qualitative analysis was conducted, in which the areas were observed bilaterally in two adjacent sections and assigned a score from 0 to 2. Zero indicated no labeled cells within the nucleus, 1 indicated very sparse staining or staining that was very light in color, and 2 indicated dark labeling or dense populations of labeled cells within the nucleus.

### Statistics

Separate two-way ANOVAs were computed for NCM, CMM, and Tn to determine whether rhythmicity of the stimulus and sex influenced the density of ZENK-immunolabeled cells within the region. A mixed model ANOVA was used for the striatum to assess whether rhythmicity and sex (between animals), as well as location within the striatum (within animals), influenced the density of ZENK-immunolabeled cells. To investigate an interaction, paired t-tests were conducted within each sex for lateral and medial striatum. All statistics were calculated using SPSS 21 (IBM, Armonk, NY).

## Results

In NCM, a significant main effect of stimulus condition was found (*F*_1,31_ = 5.73, *p* = 0.023), such that the density of ZENK-immunolabeled cells was greater in birds exposed to arrhythmic than rhythmic song (Figure 2). There was no effect of sex on density of ZENK labeled cells (*F*_1,31_ = 0.09, *p* = 0.765), and no significant interaction between stimulus condition and sex (*F*_1,31_ = 0.02, *p* = 0.885).

A significant effect of stimulus condition was also detected in CMM (*F*_1,31_ = 4.34, *p* = 0.046). As in NCM, birds exposed to arrhythmic song had an increased density of ZENK-immunolabeled cells. A main effect of sex was also detected in CMM (*F*_1,31_ = 4.55, *p* = 0.041), such that females had greater density of ZENK-immunolabeled cells than males (Figure 3). A significant interaction between rhythm condition and sex was not detected (*F*_1,31_ = 0.68, *p* = 0.414).

In Tn, a significant main effect of stimulus type was found (*F*_1,31_ = 4.64, *p* = 0.039; Figure 5). As in NCM and CMM, birds exposed to arrhythmic song had greater density of ZENK-immunolabeled cells (Figure 5). There was no effect of sex (*F*_1,31_ = 0.11, *p* = 0.737) and no interaction between rhythm condition and sex (*F*_1,31_ = 0.29, *p* = 0.596).

Unlike the other areas quantified, the striatum did not show differences between the two stimulus types – ZENK expression was equivalent across birds exposed to normal and arrhythmic songs (*F*_1,30_ = 0.23, *p* = 0.633). There was also no significant effect of sex (*F*_1,30_ = 0.39, *p* = 0.537). However, a significant difference existed between the lateral and medial striatum (*F*_1,30_ = 92.70, *p*<0.001), and this relationship was affected by sex (sex×location interaction: *F*_1,30_ = 11.94, *p* = 0.002). Expression in both males and females was greater in the medial than lateral striatum (*t*_16_ = 7.972, *p*<0.001 and *t*_16_ = –5.514, *p*<0.001 respectively), but the difference appeared much larger in males, largely due to a near absence of labeling in the lateral striatum (Area X) of males (Figure 4). There was no significant interaction between location within the striatum and stimulus condition (*F*_1,30_ = 0.54, *p* = 0.470), nor was there an interaction between sex and stimulus condition (*F*_1,30_ = 0.29, *p = *0.597). The three-way interaction among sex, location in the striatum, and stimulus condition was also not statistically significant (*F*_1,30_ = 1.43, *p* = 0.241).

**Figure 6:**
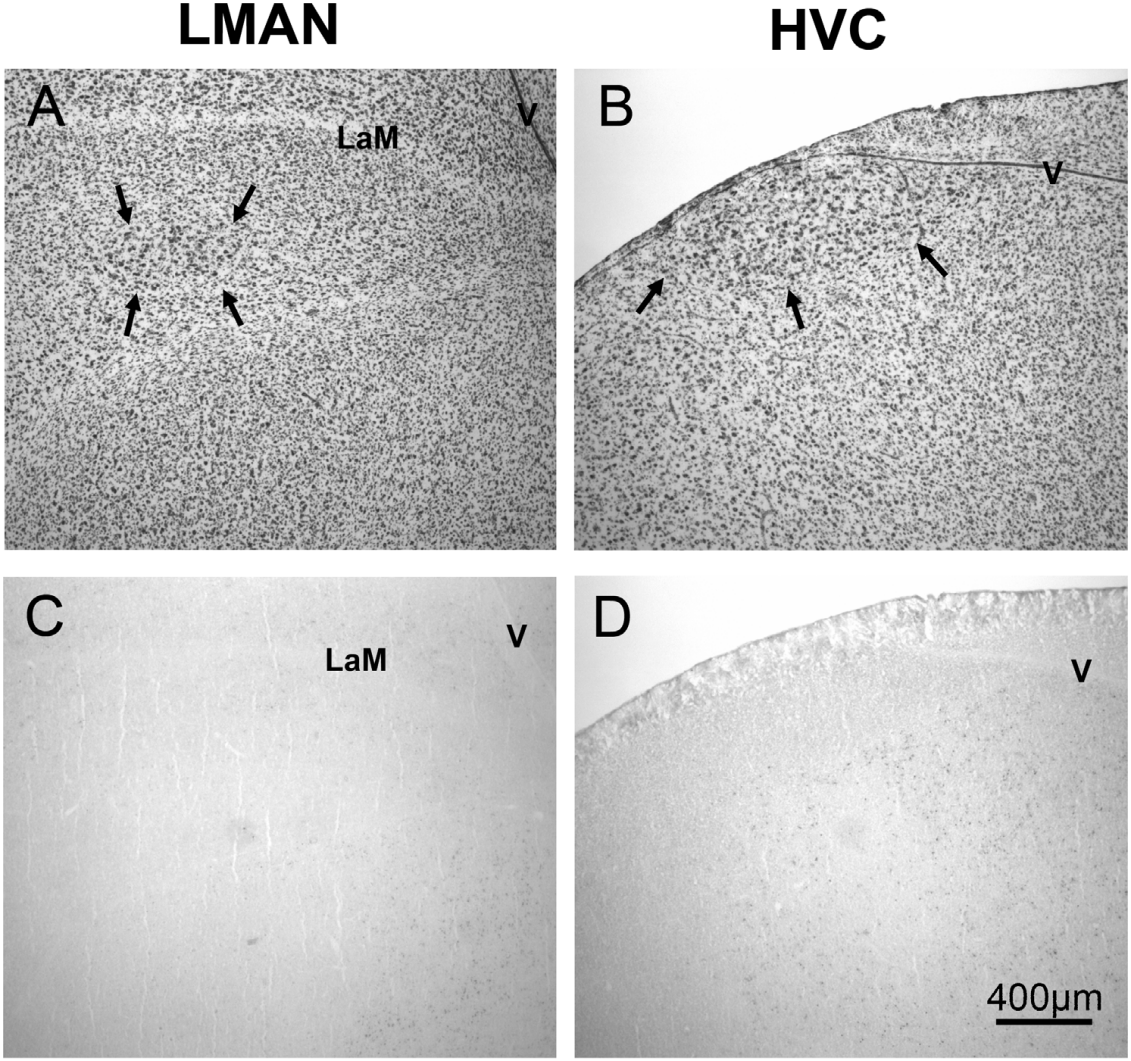
Relative absence of ZENK expressing cells in LMAN and HVC. Panels A and B depict thionin stained sections, with arrows showing borders of LMAN and HVC from a male exposed to arrhythmic song. Panels C and D depict adjacent sections with representative samples of immunohistochemical labeling. LaM = lamina mesopallialis; V = ventricle.

For LMAN and HVC qualitative scoring was done in which birds were assigned a number on a 0–2 scale, with 0 indicating no detectable ZENK, 1 indicating light or very sparse labeling, and 2 indicating dark and/or abundant staining comparable to that seen in NCM. In both LMAN and HVC, scores were very similar across the sexes and stimulus conditions and were mostly 0s (Table 1). No animals were assigned 2s for either brain region (Figure 6).

**Table 1:** Numbers of animals of each sex and stimulus condition exhibiting no or very modest labeling in two cortical song control regions, LMAN and HVC.

		LMAN		HVC	
	Score*	Rhythmic	Arrhythmic	Rhythmic	Arrhythmic
Male	0	7	7	6	7
	1	2	2	3	3
Total		9	9	9	8
Female	0	8	6	4	5
	1	1	2	2	1
Total		9	8	6	6

*0 = no detectable ZENK expression; 1 = very light or sparse labeling; 2 = dark or dense labeling (no individuals were assigned this score).

## Discussion

### Summary

The present results indicate that arrhythmic song induces greater ZENK expression in the auditory cortical areas, NCM and CMM, and the amygdala homolog, Tn, compared to un-manipulated (rhythmic) zebra finch song. Effects of stimulus type were not observed in Area X, LMAN or HVC, indicating that these differences in neural responses to song rhythmicity in the adult zebra finch are specific to the regions described. Effects associated with the sex of the animals were detected in two brain areas. First, greater ZENK expression was induced in CMM in females compared to males across stimulus groups. Second, while labeling across the sexes was increased in the medial compared to lateral striatum, the difference was greater in males due to a near absence of ZENK expression in Area X following both types of song stimuli.

### NCM and CMM

The results in NCM and CMM of increased ZENK expression with arrhythmic as compared to rhythmic song can be considered in the context of human auditory processing. In humans the auditory association cortex has increased activity in response to unexpected perturbations in one’s own speech [64]. Neurons in this area are thought to code for mismatch between expected and perceived auditory feedback [64], [65]. The pattern of results seen in NCM and CMM is consistent with data from humans, in which fMRI revealed greater activity in the secondary auditory cortex with exposure to an arrhythmic compared to a rhythmic tone sequence [66]. Thus, one possibility is that the increase in neural activity in NCM and CMM in response to arrhythmic song stems from detection of deviation from the temporal regularity expected based on the learned song template. Data on comparisons of conspecific to heterospecific song are consistent with this idea. Songs from birds other than zebra finches produce little or no expression of ZENK in both NCM and CMM, whereas conspecific song produces a robust response in these regions [42], [51]. We suggest that arrhythmic song is similar enough to natural zebra finch song so as to be detected as a (perhaps inappropriate) variant of conspecific song, whereas heterospecific song is different enough that it does not activate this system of error detection. It will now be important to determine whether auditory template formation during development is necessary for zebra finches to be sensitive to the rhythmic characteristics of conspecific song.

Exposure to reverse zebra finch song has been used as a way of testing neural response to changes in temporal pattern [67], because the total amount of song and the spectral qualities remain unchanged from normal conspecific song. However, reverse song differs from normal vocalizations in more characteristics than rhythmicity, including the onset and decline within each note and the overall structure of the bout. Reverse song induces less neural activation than other forms of conspecific song in some populations of cells within NCM [68]. These results indicate that the aspects of song altered by reversing it, including timing, bout structure and individual note dynamics, are important for neural responses to conspecific song within NCM. Thus, one possibility is that reverse song is different enough that it is not recognized as an altered form of conspecific song, and does not activate cells within NCM involved in error detection.

Untutored zebra finch song has also been used as a control stimulus; it is produced by a zebra finch and thus has similar motif structure to that of tutored song, but contains notes of unusual frequency, duration, and inflection [12]. Untutored song induces less ZENK expression than tutored zebra finch song in NCM and CMM in both juveniles [43] and adults [55]. These results differ from the current study in that aberrant song reduced ZENK expression rather than increasing it. Similar to the response to reverse song, this pattern may be due to untutored song being too dissimilar to normal song, or too inconsistent, to be detected as normal song with errors. Collectively, the results also indicate that response of auditory cortical neurons requires not just the overall motif and bout structure, but the characteristics of individual notes must be consistent with tutored zebra finch song.

While various auditory stimuli can induce different patterns of ZENK expression in the songbird brain, it is unknown whether these stimuli activate the same types of neurons. The phenotype of ZENK expressing cells was not evaluated in this study. For example, a substantial proportion of the cells in NCM are GABAergic, and these inhibitory cells can influence auditory perception [69]. Increased neural activity within NCM in response to arrhythmic compared to rhythmic song may reflect inhibitory processing rather than stimulation of a functional response. The phenotype of the ZENK+ cells should be evaluated in future studies.

It has been proposed that the auditory song template learned by juvenile zebra finches is stored in NCM [50]. This hypothesis is supported by the finding that in the template formation stage, playback of tutor song induces neuronal activity within NCM and CMM, but not other song system nuclei [70]. Song template storage within NCM is consistent with the hypothesis that NCM is involved in error detection because the site of template storage is a logical location at which to compare the template and a song example. Storage of the template within NCM would also allow NCM neurons to assess other characteristics of song, in addition to rhythm, that could influence perception of whether a sound is conspecific song and the quality of that song.

In the present study, greater ZENK induction was seen in females compared to males in CMM, specifically. Unlike in the song control system [17], sex differences in morphology of auditory structures have not been extensively described; the borders of these brain regions are not particularly distinct, and qualitatively the structure of the regions appears similar in males and females. NCM and CMM are thought to be involved in analysis of songs for purposes of mate selection in females [71]. CMM in particular is able to discriminate between directed and undirected songs [72], which is necessary for evaluating potential mate directed song quality. The increased neural activity in response to song in CMM may therefore be due to CMM being used by females for analysis of potential mates.

### Tn

Compared to NCM and CMM, much less research has been conducted regarding factors influencing neural activation and ZENK expression in Tn. A previous study in our lab demonstrated that ZENK expression in the Tn of females paired with males is positively correlated with behaviors indicative of pair bonding, including frequency of clumping with a mate as well as frequency and duration of preening [73]. ZENK expression in Tn is also positively correlated with the number of mount attempts in male house sparrows [60]. In ring doves, ZENK expression in Tn in pair bonded birds is greater than in un-bonded birds following a preference test between a mate and a novel bird [74]. In addition, the level of ZENK expression can be accurately used to predict whether the bird is pair bonded [74]. The amygdala is part of a network that controls social behavior, including sexual, parental, and aggressive behavior, in a broad range of species [75], [76]. It is not known whether the birds in this study formed pair bonds prior to being moved to single sex aviaries; if so, they were physically separated from their mates at that point. One possibility is that the increased activity in response to arrhythmic song may be part of the process of evaluating the song as indicating a poor potential partner. The phenotype of ZENK+ cells within Tn in the present study is unknown. However, given the abundance of GABAergic cells seen in the pigeon Tn [77], it is possible that arrhythmic song causes an increase in activity of inhibitory cells, potentially inhibiting selection of the singer for a mate.

An additional potential interpretation of the pattern of neural activation in Tn is suggested by human fMRI and PET studies. When participants were presented with a variety of non-speech auditory stimuli, activity in the right basolateral amygdala was positively correlated with ratings of unpleasantness of the auditory stimuli [78]. Blood flow increased bilaterally in the lateral amygdala in response to aversive sounds compared to white noise [79]. Additionally, pleasurable music leading to participants getting “chills” reduced blood flow in the amygdala bilaterally [80]. Together these studies indicate that increased activity in the amygdala is induced when auditory stimuli are perceived as aversive. The increased activity in Tn may suggest that arrhythmic song is perceived as aversive by zebra finches. This may be combined with the social interpretation, in that a song perceived as aversive may have greater salience for rejection of the singer as a potential mate.

### Striatum

In the striatum, an effect of region was detected, such that ZENK labeling was less dense in the lateral (Area X in males) compared to the medial striatum. Interestingly, this difference was greater in males than in females. These results expand on previous data from our lab in juvenile males in which conspecific and heterospecific song, as well as tones, induced a significantly lower density of ZENK labeled cells in Area X than in the medial striatum [81]. In contrast, labeling was uniform throughout the striatum in young females [81]. The current study found a difference between lateral and medial striatum in females as well as males indicating that differences in these areas in females may develop as animals get closer to maturity. Together, these studies suggest that the medial striatum is involved in processing of auditory stimuli, but not in the aspect of rhythmic discrimination assessed in this study. In addition, the low level of ZENK expression in Area X in males has been suggested to indicate a role for this brain region in song learning or production [81] rather than auditory processing, in contrast to the conclusions from some human data [66].

### Methodological Considerations

#### HVC and LAMN

Little ZENK expression was seen in either HVC or LMAN in any of the groups in the present study. While intriguing, these results do not completely exclude the possibility of neuronal activity in response to auditory stimuli in these two regions. In fact, HVC exhibits specific electrophysiological responses to a bird’s own song [58] and its tutor’s song [59], with much lower responses to general conspecific song [59]. These results are consistent with the present data which showed limited ZENK expression in these regions in response to the songs of unfamiliar zebra finches. In addition, one needs to consider that analyses of ZENK protein and electrophysiology do not always show the same pattern [54], [68]. It has been proposed that immediate early gene expression may be regulated differently in several nuclei, including HVC and LMAN, than the rest of the brain because ZENK is not expressed in these areas after presentation of stimuli that induce electrophysiological responses [40] or after treatment with a GABA antagonist [82]. Thus, while this study does not suggest a role for these areas in rhythm processing, the possibility cannot be rejected based on the present data.

#### Stimuli

Sound levels in the intervals between syllables were not identical between the rhythmic and arrhythmic stimuli. However, these differences are highly unlikely to have affected our results for several reasons. First, no significant correlations were detected between ZENK labeling and the average intensity of the intervals between syllables in either the rhythmic or arrhythmic group for any of the regions that showed an effect of stimulus type (all r<0.37, p>0.14). Second, the average power of these intervals was less than 1.2% of that of the syllables for both manipulations. Characteristics of these gaps between syllables other than their duration are therefore probably far less salient than the notes themselves. Third, the difference between the power levels of the intervals in the two stimulus types as measured in playback through the speakers is half of that in the pure stimuli (which are depicted in Figure 1). Finally, the power of the intervals was not consistently higher in either the rhythmic or arrhythmic stimulus.

### Potential Translational Implications

The zebra finch has been used previously as a model for developmental stuttering. Delayed auditory feedback can induce stuttering like syllable repetitions in zebra finches [83], [84] indicating the importance of normal auditory feedback for accurate vocalization. Helekar et al. (2003) found that 7% of the males in their colony naturally produce a stuttering-like song with single syllable repetitions, and 53% of males tutored by these repeaters also produce single syllable repetitions in their song [85]. Based on fMRI data, these birds that learn to repeat syllables have decreased responses to tutor song and increased responses to unfamiliar conspecific song in field L [86], the avian homolog of the primary auditory cortex [15]. These results suggest some dysfunction in the learning process, perhaps related to storage of an auditory template. However, activity in NCM and CMM was more variable across the syllable repeating and normal song groups, thus significant differences could not be discerned [86]. Assessment of neural responses as stuttering-like song develops would provide further understanding of specific neural mechanisms.

Animal models for many of the other disorders that involve deficits in rhythm and timing perception exist, but in these cases as well the focus is on aspects of the disorders other than timing and rhythm. For example, models of autism center around the presence of social and stereotyped behaviors in rodents [87]. Rodent models of schizophrenia are widely varied with effects on motor, cognitive and social behaviors [88]. A rat model of dyslexia with specific neurological deficits has impairment in tasks of time perception [89], but the rat as a model is restricted in its applicability to communication disorders because this species does not learn complex vocalizations. While valuable information is collected from these models, songbirds offer advantages due to specific similarities to humans. For example, they are vocal learners, undergoing critical periods of auditory and sensorimotor learning to achieve highly stereotyped yet complex adult-like songs. In addition, they rely on visual and auditory cues as opposed to olfactory, and they form monogamous pair bonds. Further study of the basis of rhythm perception and rhythm deficits is needed in animal models in order to begin developing new therapies that target the timing-based deficits observed in this broad range of disorders.

In sum, zebra finches are an excellent potential model for studying neural mechanisms underpinning human rhythm perception and its relation to speech and language processing. This avian species provides a model through which neurochemical mechanisms of rhythm perception and dysfunction can be tested to gain a deeper understanding of rhythm processing for application to both healthy and disordered human development. The present study has shown that NCM, CMM, and Tn increase neural activity in response to arrhythmic song, indicating a role for rhythm in auditory discrimination and social behavior such as mate choice in the zebra finch. Further studies are needed to understand the development and mechanisms underlying neural responses to rhythm.
